# Feasibility and acceptability of home-based HIV testing among refugees: a pilot study in Nakivale refugee settlement in southwestern Uganda

**DOI:** 10.1186/s12879-018-3238-y

**Published:** 2018-07-16

**Authors:** Kelli N. O’Laughlin, Wei He, Kelsy E. Greenwald, Julius Kasozi, Yuchiao Chang, Edgar Mulogo, Zikama M. Faustin, Patterson Njogu, Rochelle P. Walensky, Ingrid V. Bassett

**Affiliations:** 1 Department of Emergency Medicine, Brigham & Women’s Hospital, 75 Francis Street, Boston, MA 02115 USA; 2 Medical Practice Evaluation Center, Department of Medicine, Massachusetts General Hospital, 50 Staniford St, 9th Floor, Boston, MA 02114-2698 USA; 3 Harvard Medical School, Boston, MA 02114-2698 USA; 4 Harvard Humanitarian Initiative, Cambridge, MA USA; 5 Division of General Medicine, Massachusetts General Hospital, 50 Staniford St, Suite 560, Boston, MA 02114-2698 USA; 6 Harvard Affiliated Emergency Medicine Residency, 75 Francis Street, Boston, MA 02115 USA; 7 United Nations High Commissioner for Refugees, Representation in Uganda, P.O. Box 3813, Kampala, Uganda; 8 Department of Community Health, Mbarara University of Science and Technology, P.O Box 1410, Mbarara, Uganda; 9 Bugema University, Kasese Campus, P.O. Box 6529, Kampala, Uganda; 10 United Nations High Commissioner for Refugees, Representation in Kenya, P.O. Box 43801-00100, Nairobi, Kenya; 11 Division of Infectious Disease, Massachusetts General Hospital, 50 Staniford St, 9th Floor, Boston, MA 02114-2698 USA; 12 Division of Infectious Disease, Brigham & Women’s Hospital, 50 Staniford St, 9th Floor, Boston, MA 02114-2698 USA; 13 Harvard University Center for AIDS Research (CFAR), 50 Staniford St, 9th Floor, Boston, MA 02114-2698 USA

**Keywords:** HIV testing, Home-based HIV testing, Refugees, Uganda, Displaced population, Humanitarian, HIV

## Abstract

**Background:**

Refugees in sub-Saharan Africa face both the risk of HIV infection and barriers to HIV testing. We conducted a pilot study to determine the feasibility and acceptability of home-based HIV testing in Nakivale Refugee Settlement in Uganda and to compare home-based and clinic-based testing participants in Nakivale.

**Methods:**

From February–March 2014, we visited homes in 3 villages in Nakivale up to 3 times and offered HIV testing. We enrolled adults who spoke English, Kiswahili, Kinyarwanda, or Runyankore; some were refugees and some Ugandan nationals. We surveyed them about their socio-demographic characteristics. We evaluated the proportion of individuals encountered (feasibility) and assessed participation in HIV testing among those encountered (acceptability). We compared characteristics of home-based and clinic-based testers (from a prior study in Nakivale) using Wilcoxon rank sum and Pearson’s chi-square tests. We examined the relationship between a limited number of factors (time of visit, sex, and number of individuals at home) on willingness to test, using logistic regression models with the generalized estimating equations approach to account for clustering.

**Results:**

Of 566 adults living in 319 homes, we encountered 507 (feasibility = 90%): 353 (62%) were present at visit one, 127 (22%) additional people at visit two, and 27 (5%) additional people at visit three. Home-based HIV testing participants totaled 378 (acceptability = 75%). Compared to clinic-based testers, home-based testers were older (median age 30 [IQR 24–40] vs 28 [IQR 22–37], *p* < 0.001), more likely refugee than Ugandan national (93% vs 79%, < 0.001), and more likely to live ≥1 h from clinic (74% vs 52%, < 0.001). The HIV prevalence was lower, but not significantly, in home-based compared to clinic-based testing participants (1.9 vs 3.4% respectively, *p* = 0.27). Testing was not associated with time of visit (*p* = 0.50) or sex (*p* = 0.66), but for each additional person at home, the odds of accepting HIV testing increased by over 50% (OR 1.52, 95%CI 1.12–2.06, *p* = 0.007).

**Conclusions:**

Home-based HIV testing in Nakivale Refugee Settlement was feasible, with 90% of eligible individuals encountered within 3 visits, and acceptable with 75% willing to test for HIV, with a yield of nearly 2% individuals tested identified as HIV-positive.

## Background

Sub-Saharan Africa accounts for 70% of people living with HIV globally and the highest burden of HIV transmissions and HIV/AIDS related mortality in the world [1]. Refugees, 3.5 million of whom live in Sub-Saharan Africa, are particularly susceptible to contracting HIV because of threats of sexual violence, and increased vulnerability due to stress and inadequate nutrition [2–5]. Refugees experience substantial obstacles accessing HIV testing, including having to prioritize day-to-day survival such as food, safety, and shelter over their future health [6]. Additionally, in a routine clinic-based HIV testing study in Nakivale Refugee Settlement in Uganda, refugees who lived further from clinic had an increased likelihood of testing HIV-positive than those who lived one hour or less from clinic [7]. Strategies that increase the ease of accessing HIV testing for those living in refugee settlements will likely result in decreased morbidity, mortality and transmission of disease.

Home-based HIV testing has been effective in many resource limited settings in sub-Saharan Africa [8–20], but may not be a successful testing strategy in a refugee settlement. Many refugees have endured violent conflict and/or sexual violence [5, 21], and thus may be less willing to accept strangers into their homes. While the impact of humanitarian crisis on mental health is broad [22, 23], some refugees suffer from non-disordered psychological distress and post-traumatic stress disorder [24], which may limit their willingness to accept home-based HIV testing. Counselors conducting home-based testing may be from a different country than the participant [25], which could result in further distrust. Refugees in settlements often live physically close to others [26], with little space in their own home and nearby other homes, and may therefore have heightened fear of disclosure of their HIV status and concern regarding potential stigmatization and harassment due to an HIV diagnosis. Further, with numerous livelihood challenges in refugee settlements [6, 26–28], it is possible that refugees are not easily encountered at home as they may be away seeking employment or cultivating their land to grow food.

To assess the feasibility and acceptability of home-based HIV testing in a refugee setting, we conducted a feasibility and acceptability pilot study in Nakivale Refugee Settlement in southwestern Uganda. We compared demographics of home-based and clinic-based HIV testers from a previous routine clinic-based HIV testing study in Nakivale [25] to discern if a unique population was reached by home-based HIV testing.

## Methods

### Study setting

This pilot was conducted in Nakivale Refugee Settlement, a 71-mile^2^ settlement established in 1960, located in rural southwestern Uganda, and managed by the United Nations High Commissioner for Refugees (UNHCR) and the Ugandan government [26]. At the time of this study in 2014, Nakivale was home to 68,000 refugees, many of whom had lived in the settlement for generations; 52% of the settlement’s population was from the Democratic Republic of the Congo (DRC), 17% from Somalia, 15% from Rwanda, and 15% from Burundi [29]. The settlement is divided geographically into 79 villages with an average of 800 to 1000 people per village [26], and with villages largely comprised of refugees from the same country of origin. Refugees are provided plots of land of 50 m by 100 m on which to build their own home and grow food or raise animals [26]. Ugandan nationals also live in and around Nakivale and access health services in the settlement. Medical Teams International (MTI), the non-governmental organization responsible for health-related activities in Nakivale, oversees the medical care provided at the four health facilities in the settlement. At these health centers, HIV testing is offered free of charge and is conducted using serial rapid HIV tests outlined in the Uganda national guidelines [30]. At the time of this study, the serial HIV test algorithm started with Determine HIV-1/2 Ag/Ab Combo [31], confirmed positive specimens using HIV 1/2 STAT-PAK Assay [32], and used Uni-gold HIV as a tiebreaker when needed [33]. Additionally, antiretroviral therapy (ART) is available free of charge in these locations and, at the time of this study, was offered to those with a CD4 ≤ 350/mm^3^ or World Health Organization (WHO) stage III/IV based on 2010 WHO guidelines [34]. Data from a routine clinic-based HIV testing study conducted at Nakivale Health Center in 2013 showed the new HIV diagnoses frequency was 3.3% in the standard of care period and 4.5% in the intervention period (*P* > 0.5) in Nakivale [25], and the Uganda AIDS indicator survey reported HIV prevalence in the surrounding region of rural Uganda to be 7.3% [35].

### Study design

From February–March of 2014, research assistants spent three weeks conducting home visits in Nakivale. Each week they visited a distinct village (labeled Village 1–3 for the purposes of this study). Village 1 is considered by locals in Nakivale to be Congolese, Village 2 Burundian and Village 3 Rwandan. Despite this perception, each village has people from various countries of origin including Ugandans. Research assistants were fluent in English, Kiswahili, Kinyarwanda, and Runyankore, had prior experience in HIV counselling, and had been trained in rapid HIV testing by the Center for Disease Control and Prevention (CDC) in Uganda. Working in teams of two, research assistants visited consecutive homes covering all dwellings in each village. Each home received a total of three separate visits, on alternative days of the week at different times of day, unless all adults were present at an earlier visit or unless the research assistants were asked not to return. Research assistants gathered information from those at home and from neighbors about how many individuals lived in each home and the approximate age of each person to evaluate potentially eligible participants. HIV counseling and testing were offered to all adults in residence who met eligibility criteria. Research assistants consented participants and offered an HIV test and an oral survey in one of the four languages noted above. Survey information, which was collected directly onto an electronic tablet, included demographic information (i.e. sex, age, refugee status, years in Nakivale, country of origin, relationship status, education), HIV knowledge, HIV testing history, and approximate travel time to clinic. Research assistants used serial rapid HIV tests as per Ugandan national guidelines [30]. The HIV tests used included Determine™ HIV-1/2 as a screening test [31], HIV 1/2 Stat-Pak™ Assay as a confirmatory test [32], and Uni-gold ™ HIV as a tiebreaker; these are the same HIV tests used at Nakivale Health Center at the time of this study [33]. Participants identified as HIV-positive were referred to the nearest health center’s HIV clinic with instructions to attend clinic within 1 week.

### Protecting confidentiality

To protect participant privacy during data collection, an attempt was made to find a confidential location in or around the homes – participants were asked where they would feel safe taking the test and survey. To protect data confidentiality, survey data were de-identified and stored in electronic, pass-code protected tablets and on computers with anti-virus software. No individual identities were used in any reports or publications that resulted from this study.

### Subject selection

Three distinct villages in Nakivale were selected for the home-based HIV testing pilot. This sampling plan was designed to include different villages representing predominant country groups in the settlement: Congolese, Burundi, and Rwandan. Additionally, Ugandan nationals reside throughout Nakivale, and we anticipated – based on findings from our clinic-based Nakivale study -- that our sampling plan would include nationals. Though Somalis were also a predominant country group in Nakivale (17% of settlement population) [29], < 4% of individuals identified as HIV-positive in our routine clinic-based HIV testing study were from Somalia [7]; therefore, we did not conduct home-based HIV testing in a Somali village in this pilot study. Eligibility criteria included: adults ≥18 years of age, capacity to give informed consent, and the ability to speak English, Kiswahili, Kinyarwanda, or Runyankore, similar to the inclusion criteria for our previous study in Nakivale [25]. The clinic-based HIV testing comparison cohort participated in routine HIV testing at Nakivale Health Center III in 2013 [25].

### Classification of endpoints/statistical analysis

Our primary goals were to assess the feasibility and acceptability of home-based HIV testing in Nakivale, and to compare characteristics of home-based and clinic-based testers in Nakivale. To assess feasibility, we evaluated the proportion of eligible individuals encountered at home. Additionally, we assessed the proportion of eligible individuals found at each household on initial and subsequent visits. To assess the acceptability of home-based HIV testing in the refugee settlement, we evaluated the proportion of eligible individuals at home who participated in home-based HIV testing. We compared the demographics of the home-based testing participants to clinic-based testing participants who lived in Nakivale assessing sex, age, refugee status, years in Nakivale, country of origin, marital status, education, HIV knowledge, HIV testing history, distance to clinic and HIV test result. Participant characteristics were reported as frequency (percent) or median with interquartile range (IQR), as appropriate and compared between home-based and clinic-based testers using Pearson’s chi-square tests for categorical variables and Wilcoxon rank sum tests for continuous variables. Missing data were excluded from the analysis for variables with a small amount of missing data (< 3%). For “years in Nakivale”, those with missing data were categorized as “unknown.” Since limited information was available for those who were not present and for those who did not participate in HIV testing, we used logistic regression models with the generalized estimating equations approach to assess the effect of time of visit, sex, and number of eligible individuals present in the household on willingness to test while accounting for clustering within households. Results with two-sided *p* < 0.05 were considered statistically significant. All statistical analyses were performed using SAS 9.4 (Cary, NC, USA).

### Ethics approval and consent to participate

This study was approved by the Makerere University School of Health Sciences Institutional Review Board (Kampala, Uganda; Ref No 2012–020), the Uganda National Council of Science and Technology (Kampala, Uganda; HS 1167), and the Partners Human Research Committee (Boston, MA, USA; 2010P001963/BWH). Written consent was obtained from all surveyed and tested study participants.

## Results

### Feasibility and acceptability of home-based HIV testing

Over the 3-week period, we visited 319 homes in three villages. Most households consisted of ≥2 eligible people and 9% of the households were abandoned (Table 1). For feasibility, we encountered 507 (90%) of the 566 eligible individuals reported to be living in these homes within three visits. For acceptability, 378 (75%) of the 507 encountered individuals participated in home-based HIV testing and received their test results. The proportion of those at home who tested for HIV varied by village (Village 1: 89%, Village 2: 64%, Village 3: 70%; *p* < 0.001). The majority (480/507, 95%) of the individuals reached were encountered during visits 1 and 2. There was a small decrease in the proportion willing to test from visit 1 to visit 2 (77 to 73%), and a steep decline on visit 3 (44%). Three individuals at home during visit 1 did not agree to testing initially, but agreed to and consented during a subsequent visit—two of these individuals tested during visit 2 and one during visit 3. All others tested during the visit in which they were first encountered at home. Of the 378 who tested for HIV, 7 (1.9%) were diagnosed HIV-positive (Village 1: 4 [2.5%], Village 2: 3 [2.9%], Village 3: 0 [0%]). Among 233 households with > 1 eligible individual, 119 households had more than one person participate in HIV testing and 114 of those households (96%) had sero-concordance identified in the study within the household.

**Table 1:** Household Data for Home-Based HIV Testing Participants

Variable	Overall	Village 1	Village 2	Village 3
Country of origin of majority of participants		DRC^a^	Burundi	Rwanda
(% of HIV testing participants in village from this country)		78	71	74
Number households visited	319	108	114	97
Eligible individuals in households	566	206	184	176
Size of household, N (% of total households)				
0	27 (9)	10 (9)	12 (11)	5 (5)
1	59 (19)	11 (10)	28 (25)	20 (21)
2	203 (64)	71 (66)	68 (60)	64 (66)
3	21 (7)	12 (11)	4 (4)	5 (5)
4	7 (2)	3 (3)	2 (2)	2 (2)
5	2 (1)	1 (1)	0 (0)	1 (1)
Encountered at home, N (% of eligible)	507 (90)	181 (88)	162 (88)	164 (93)
Visit 1	353 (62)	119 (58)	112 (61)	122 (69)
Visit 2	127 (22)	56 (27)	43 (23)	28 (16)
Visit 3	27 (5)	6 (3)	7 (4)	14 (8)
HIV Tested, N (% of encountered)	378 (75)	161 (89)	103 (64)	114 (70)
Visit 1	272 (77)	108 (92)	79 (71)	85 (70)
Visit 2	93^b^ (73)	49^b^ (84)	24 (56)	20 (71)
Visit 3	13^c^ (44)	4^†^ (57)	0 (0)	9 (64)
HIV-positive, N (% of tested)	7 (1.9)	4 (2.5)	3 (2.9)	0 (0)

^a^DRC = The Democratic Republic of the Congo

^b^2 testers encountered in visit 1 but consented to testing in Visit 2

^c^1 tester encountered in visit 1 but consented to testing in Visit 3

### Characteristics of home-based vs. clinic-based HIV testing participants

There was no significant difference in sex distribution among home-based and clinic-based testing participants (56% vs. 53% female respectively, *p* = 0.20), but home-based testers were slightly older (median age 30 [IQR 24–40] vs. 28 [IQR 22–37], *p* < 0.001), were more often refugee than Ugandan national (93% vs. 79%, *p* = <0.001), and had lived in Nakivale longer (median time of 6 years [IQR 3–8] vs. 4 years [IQR 1–8], p < 0.001, Table 2). Of the participants who were tested, more home-based testers were from the Democratic Republic of the Congo (35% vs. 25%) or from Burundi (24% vs. 15%). A greater proportion of home-based testers were married/living together (77% vs. 63%). Fewer home-based testers had some secondary school or above education than clinic-based testers (11% vs. 20%). Home-based HIV testers had a higher proportion that scored ≥75% correct on the 4 question HIV knowledge test (70% vs. 60%) but no significant difference in the proportion with previous HIV tests (78% vs. 74%) or in the proportion with HIV tests within the past year (40% vs. 36%). Home-based HIV testers were more likely to live ≥1 h from a health clinic (74% vs. 52%). The yield for HIV-positive test results was not significantly different between home-based and clinic-based testers (1.9 vs. 3.4%, *p* = 0.27).

**Table 2:** Comparison of home-based and clinic-based HIV test participants

Variable	Home-based testers *N*=378 N (%)	Clinic-based testers *N*=6443 N (%)	*P*-value*
Female	212 (56)	3395 (53)	0.20
Age category			< 0.001
18–24	112 (30)	2340 (36)	
25–34	116 (31)	2172 (34)	
35–44	81 (21)	1178 (18)	
>45	69 (18)	753 (12)	
Refugee status			< 0.001
Refugee	353 (93)	5103 (79)	
Ugandan national	23 (6)	1330 (21)	
Years in Nakivale			< 0.001
<1year	7 (2)	754 (12)	
1–5years	130 (34)	2409 (37)	
≥5years	233 (62)	2670 (41)	
Unknown	8 (2)	610 (10)	
Country of origin			< 0.001
Uganda	23 (6)	1342 (21)	
Rwanda	129 (34)	2371 (37)	
DRC	134 (35)	1580 (25)	
Burundi	91 (24)	986 (15)	
Other^a^	1 (0.3)	164 (2.5)	
Relationship status			< 0.001
Married/living together	292 (77)	4053 (63)	
Single	39 (10)	1658 (26)	
Divorced/separated/widowed	47 (12)	723 (11)	
Education			< 0.001
No school	96 (25)	1330 (21)	
Some/completed primary school	239 (63)	3794 (59)	
Some secondary school and above^b^	43 (11)	1309 (20)	
HIV knowledge^c^			< 0.001
<75% correct	113 (30)	2609 (41)	
≥75% correct	265 (70)	3834 (60)	
Previous HIV test	294 (78)	4751 (74)	0.072
HIV tested within the past year	151 (40)	2308 (36)	0.10
Time to clinic			< 0.001
<1h	89 (24)	3099 (48)	
≥1h to clinic	278 (74)	3344 (52)	
HIV-positive	7 (1.9)	217 (3.4)	0.27

*Abbreviations: DRC* Democratic Republic of the Congo

*P-value based on Pearson chi-square test

^a^Other includes Somalia, Eritrea, Ethiopia, Sudan, Kenya, Tanzania, Senegal, and Zaire

^b^Includes some/completed secondary school, vocational school, certificate program, bachelors, and post graduate

^c^Questionnaire included 4 questions [correct answer]: (1) Do you think that a healthy-looking person can be infected with HIV, the virus that causes AIDS? [Yes]; (2) Can a person get HIV by sharing a meal with someone who is infected? [No]; (3) Can a pregnant woman infected with HIV or AIDS transmit the virus to her unborn child? [Yes]; (4) Can a woman with HIV or AIDS transmit the virus to her newborn child through breastfeeding? [Yes]

### Factors associated with willingness to participate in home-based HIV testing

We examined the association between a limited number of available factors and willingness to test in home-based HIV testing (Table 3). Time of visit was not significantly associated with willingness to test (*p* = 0.50). There was also no difference in willingness to test between females and males (*p* = 0.66). However, as the number of eligible individuals at home when testing was offered increased, willingness to participate increased (70% when no one else present, 73% when only one other person was present, and 86% when more than one other person was present). For each additional person present, the odds of testing increased by over 50% (OR: 1.52, 95%CI 1.12–2.06, *p* = 0.007).

**Table 3:** Predictors of willingness to participate in home-based testing, includes visits 1–3

Variable	At home, N	HIV tested, N (%)	*P* value*
Time of Visit			0.50
10am-12pm	128	101 (79)	
12pm–2pm	81	60 (74)	
2pm–4pm	195	138 (71)	
After 4pm	101	78 (77)	
Sex			0.66
Female	277	212 (77)	
Male	217	166 (77)	
Number of individuals in household present			0.007
1	105	73 (70)	
2	324	238 (73)	
3–5^a^	78	67 (86)	

*P-value from logistic regression model with all three factors included in the model

^a^Number of individuals analyzed as a continuous variable in the logistic regression model

## Discussion

To our knowledge, this is the first study to assess the feasibility and acceptability of home-based HIV testing in a refugee settlement in sub-Saharan Africa. We found that by visiting homes up to three times, we encountered 90% of eligible adults noted to be living in those households, confirming this approach is feasible. Furthermore, 75% of eligible adults encountered participated in HIV testing and received their results, reflecting the acceptability of home-based HIV testing among this population (Table 1). This study demonstrates that home-based HIV testing in Nakivale Refugee Settlement is possible and is a strategy that should be considered for other refugee settlements in Uganda and in nearby countries in sub-Saharan Africa.

There were numerous reasons to suggest that going to homes and encountering individuals at home might be a challenge in this context. Nakivale is geographically large, covering 71-mile^2^ [26], and we were not certain if our study team would successfully reach all the households in these remote villages while carrying HIV testing supplies. However, by allowing for 1–2 h of driving in the morning and late afternoon and equipping each team of research assistants with backpacks with all necessary research and HIV testing supplies (i.e. electronic tablet, gloves, HIV test kits, cleaning supplies), our teams moved about with ease. Further, though the refugee settlement is expansive and refugees are initially given large plots of land, the dwellings in each village are close to one another, often with only approximately 3 m of land separating homes. This is because many refugees choose to live near small village trading centers rather than on their land; they frequently travel 1–2 h walking to cultivate their land allotment. The layout of the villages, with homes mostly lined up along both sides of a main street, made it possible for research staff to move quickly between homes and to draw maps of each village that enumerated households to facilitate multiple visits when necessary. By returning to homes up to three times, we encountered most adults living in each household.

Aware that many refugees in Nakivale previously experienced violent conflict and sometimes gender-based violence as a weapon of war [36–39], we had concern that research staff might not be welcomed into the villages or into homes in the refugee settlement. Further, given the proximity of neighboring homes, we thought that fears regarding potential lack of confidentiality of HIV test results might compound this problem. This was not the case. As we moved door-to-door, people living in the village volunteered information about how many people lived in each home, how old their neighbors were, and which homes were abandoned. This acceptance of our research staff in the villages may have been facilitated by the demographics of our research staff (individuals from the DRC, Rwanda, and Burundi), all refugees from similar countries of origin as those in the villages and able to communicate in the native language of many of our participants. Many of the adults who we did not encounter were reported to be far away for an extended period, working elsewhere in Uganda.

We identified differences comparing home-based HIV testers to clinic-based testers living in Nakivale, some of which are potentially explained by the structure of the refugee settlement. Home-based testers were older, with a difference in median age of 2 years. It may be that younger individuals move from the village to live closer to “basecamp”, the social and economic center of Nakivale. In basecamp there are informal stores based out of windows of homes (including electronic stores for charging cellular phones), small motels providing room and board for travelers, merchants with tarps spread on the ground selling goods (i.e. jerrycans, washing bowls, shoes, and maize), and a small selection of restaurants and bars. Similarly, Ugandan nationals living in Nakivale likely choose to live closer to the center of the settlement where commerce is most active and where Nakivale Health Center is located. It is likely for this reason that home-based testing had a higher proportion of refugee vs. Ugandan national participants compared to clinic-based testers (93% vs. 79% respectively). It may also be that refugees are less willing to access clinic-based services such as HIV testing, which are provided mostly by Uganda national staff who are often not facile in their native language. Not surprisingly, home-based testers were more likely to live further from a health clinic. This may be because of the remote location of the 3 villages where we conducted the pilot, though there are 4 health clinics distributed around Nakivale. More likely, given the scarcity and cost of transportation in Nakivale, clinic-based HIV testers simply lived closer to and more often accessed clinic.

Home-based testers lived in Nakivale longer than clinic-based testers, with more than half reporting living in Nakivale ≥5 years (62% vs. 41%), and a minority living in Nakivale for < 1 year (2% vs. 12%). This likely reflects how villages are established in Nakivale. As refugees are transported from transition camps elsewhere in Uganda, they are placed in new villages mostly with others from the same country of origin. By sampling villages further from basecamp, we may have unknowingly selected participants who had spent more time in Nakivale rather than new arrivals that could have been settled in villages closer to basecamp. It also may be that those with less time in Nakivale have ailments that bring them to clinic more frequently or that those who arrived more recently must depend more on clinic services than well-established refugees who have a larger network of family and friends on which to rely.

More home-based testers reported they had no schooling (25% vs. 21%) and less reported attending some secondary school and above (11% vs. 20%). This may be because of the limited number of primary schools scattered around the settlement and the reality that many children living in the villages are expected to help with essential chores such as cooking and farming [6]. Additionally, there is only one secondary school in Nakivale, which is located 1–3 h walking distance from the villages where our pilot took place. More likely, however, refugees did not attend school in their country of origin because of war and unrest. Additionally, individuals with more education probably sought medical care at a health center more often. Despite less schooling, home-based testers were more likely to achieve ≥75% correct answers on a 4 question HIV knowledge test (70% vs 60%), perhaps reflecting the success of prior HIV prevention campaigns in the settlement.

Even when encountered at home, it was uncertain if home-based HIV testing would be acceptable to this unique population living in the refugee settlement. This was particularly concerning given the reality that some refugees have experienced potentially traumatic events and may have posttraumatic stress disorder (PTSD) [40–42]. However, 75% of individuals encountered accepted the home-based HIV testing intervention, with the large majority (95% of participants tested) doing so during the first or second visit. Many even anticipated our visit to their home and planned to be present, telling us they usually leave to work their land during the day but instead stayed home when they heard about the home-based HIV testing services from others in the village. Given the low proportion of remaining eligible individuals willing to test, and the yield of HIV-positive results declining with each subsequent visit, limiting home-based HIV testing interventions to two home visits appears to be sufficient in this setting. While we originally thought HIV testing would be more acceptable to individuals at home alone as they would have more privacy, the odds of participating in HIV testing instead increased with each additional person home. It may be that refugees sought support from their family or permission from their partner prior to testing, and therefore could proceed with testing when those additional people were present. A campaign to notify individuals of upcoming home-based testing in their village and to invite them to be home at a specific time may facilitate uptake of HIV testing [43, 44]. Though we intentionally conducted repeat visits at different times of day, we did not find a time of day when more people tested for HIV.

While home-based testing in the refugee settlement was acceptable for most, there may be subsets of the population that found testing at home less acceptable. Other than sex, the characteristics of those who were eligible but did not test were not identified and therefore cannot be evaluated. In Village 3, comprised mostly of Rwandans, only 70% of individuals encountered where willing to test for HIV and no individuals tested HIV-positive. This was unusual considering 17% (56/330) of those found to be HIV-positive in our clinic-based HIV testing study in Nakivale were Rwandan [7]. It may be that higher risk individuals declined participation or avoided being encountered at home.

Given financial constraints, and the need to prioritize health expenditures to maximize benefits, consideration of the cost-effectiveness of various home-based HIV testing strategies in humanitarian settings is crucial. A cost-effectiveness analysis comparing home-based and facility-based testing strategies in rural South Africa found that home-based testing led to higher uptake and was less costly per client tested [45]. While knowledge of HIV status is important in HIV prevention, with resource constraints it may be more useful to evaluate HIV positivity. Though HIV-positive test results were not significantly different between home-based and clinic-based testers in Nakivale (1.9 vs 3.4%, *p* = 0.27), it is intuitive that HIV testing outside of the clinic may have a lower diagnostic yield. However, a cost-effectiveness study in Uganda comparing home-based and facility-based HIV testing which measured effectiveness as number of HIV sero-positive clients identified found that home-based testing was the least costly strategy both for the number of clients tested and for the number of positive clients identified [46]. Finally, another study in Uganda compared four HIV testing strategies (stand-alone, hospital-based, household-member, and door-to-door), and found home-based HIV testing strategies reach populations with low rates of prior testing and identified people with HIV with higher CD4 cell counts [9]. Stakeholders caring for humanitarian-affected populations will need clear goals to select appropriate HIV testing strategies for their target population. Multiple testing strategies may be needed to meet UNAIDS goals, improve the health of individuals, and curb transmission of disease in this unique setting.

This study should be viewed in the context of certain limitations. There may be an increased risk of coercion to test or to disclose one’s status to family members or others in the household with home-based testing. Despite this possibility, our study team observed the opposite. Individuals may be less likely to link to HIV clinical care following home-based testing [20, 47], as newly diagnosed individuals cannot easily be personally escorted to HIV clinic for immediate linkage as can be achieved during clinic-based testing. Follow-up data collection was beyond the scope of this pilot study; therefore, we were unable to evaluate linkage to care after home-based testing based on these data. The study was performed in three distinct geographic villages in Nakivale, representing participants from three primary countries of origin. Findings may not be generalizable to other country groups or to refugee settlements with people from different countries of origin. However, as of the end of 2016, there were nearly 1 million refugees in Uganda and numerous refugee settlements in the southern half of Uganda with similar population demographics [48, 49]. It is important to note that we do not have complete data to present sero-concordance among household members; the numbers we presented are an estimate based on existing data. Further, we did not design our data collection tools to record couples, and therefore we are unable to evaluate sero-concordance among couples. Finally, the study design comparing home-based testers from early 2014 and clinic-based testers from 2013 did not account for potential secular trends that may have effected HIV testing behaviors.

## Conclusion

Home-based HIV testing in refugee settlements may be a useful strategy to help this unique and vulnerable population know their HIV status, particularly contributing to the first 90 of the UNAIDS 90–90-90, helping to diagnose 90% of all people living with HIV [50]. Findings from this study indicate that home-based testing is not only feasible and acceptable in Nakivale Refugee Settlement in Uganda, but may reach a population that is currently underserved by clinic-based testing. Home-based testing can reduce barriers and expand access to HIV testing for hard-to-reach refugee communities. Such interventions can enhance early diagnosis of HIV infection, improve clinical outcomes, and reduce HIV transmission among refugee populations -- each critical steps toward curbing the global HIV epidemic.

## Abbreviations

ART: Antiretroviral therapy
CDC: Centers for Disease Control and Prevention
DRC: The Democratic Republic of the Congo
MTI: Medical Teams International
UNHCR: United Nations High Commissioner for Refugees
WHO: World Health Organization
